# Molecular (*cox*1), geographical, and host record investigation of monogeneans *Mazocraes australis* (Mazocraeidae), *Polylabris sillaginae*, and *P. australiensis* (Microcotylidae)

**DOI:** 10.1007/s00436-022-07664-8

**Published:** 2022-10-04

**Authors:** Md. Shafaet Hossen, Diane P. Barton, Skye Wassens, Shokoofeh Shamsi

**Affiliations:** 1 School of Animal and Veterinary Sciences & Graham Centre for Agricultural Innovation, Charles Sturt University, Wagga Wagga, NSW 2678 Australia; 2 Department of Fisheries Biology and Genetics, Bangladesh Agricultural University, Mymensingh, 2202 Bangladesh; 3 School of Environmental Sciences & Institute of Land, Water and Society, Charles Sturt University, Albury, NSW 2640 Australia

**Keywords:** Fish, Monogenea, Mazocraeidae, Microcotylidae, Molecular identification, Australia

## Abstract

This study determines the occurrence and molecular characterisation of Monogenea from three commercially important Australian fish: Australian sardine *Sardinops sagax* (Jenyns), Australian anchovy *Engraulis australis* (White), and eastern school whiting *Sillago flindersi* McKay. Earlier studies have provided only morphological species identification, whereas this study combines both morphological and molecular methods. A total of 247 fish across 3 species, sourced from the New South Wales and Victorian coasts, were examined for Monogenea. A total of 187 monogenean parasites were recovered from the gills. The overall prevalence, mean intensity, and mean abundance were 34%, 2.23, and 0.78, respectively*.* The parasites were initially classified morphologically as three species across two families. Family Mazocraeidae was represented by *Mazocraes australis* Timi et al. J Parasitol 85:28–32, 1999, and family Microcotylidae by *Polylabris sillaginae* (Woolcock, Parasitology 28:79–91, 1936) Dillon, Hargis, and Harrises, 1983 and *P. australiensis* Hayward, 1996. Molecular identification of parasites was conducted through sequencing of the mitochondrial cytochrome *c* oxidase subunit 1 (*cox*1) gene. The fish hosts in the present study were also barcoded (mitochondrial *cox*1 gene) to confirm specific identities. There was no comparable *cox*1 sequence available in GenBank for the parasites found in the present study. However, the phylogenetic tree clustered the monogenean species identified in this study according to their familial groups of Mazocraeidae and Microcotylidae. The presence of *M. australis* on *E. australis* and *S. sagax* was confirmed in this study. *Polylabris australiensis* was only found on *S. sagax* but *Si. flindersi* was found to be a host for both *Polylabris* species. This study is the first to explore the mitochondrial *cox*1 genes of these three-monogenean species. These findings will serve as a foundation for future monogenean research in Australian waters and elsewhere.

## Introduction

Monogenea are generally ectoparasitic platyhelminths that live on the body surfaces, fins, head, gills, eyes, and oral and branchial cavities depending on the species (Whittington and Chisholm 2008). In general, these worms cause severe damage to the gills due to the invasiveness of suckers, clamps, and hooks at the site of attachment (Hutson et al. 2007; Whittington and Chisholm 2008). Serious pathology and marked pathogenicity leading to the death of fish from Australian waters have previously been reported (Deveney et al. 2001). Approximately, 3500 monogenean species have been described worldwide from marine fish species (Rohde 2005) and a total of at least 316 monogenean species have been identified from various Australian fish hosts (ABRS 2009).

The Australian sardine *Sardinops sagax* (Jenyns) is a small, schooling pelagic fish of the family Clupeidae (Stewart et al. 2010) which inhabits a depth range of 0–200 m (Froese and Pauly 2019). This species is distributed all along the waters of the continental shelves of Australia and New Zealand (Fletcher 1990; Hoedt and Dimmlich 1995). *Sardinops sagax* is a planktivorous fish, which as juveniles or young feed on zooplankton such as copepods and as mature fish on phytoplankton (Froese and Pauly 2019). This fish is extensively used as a live bait food for aquaculture fish, pet food as well as human food. Therefore, *S. sagax* is considered a valuable ‘target fish’ because of its growing market demand (Stewart et al. 2010).

The Australian anchovy *Engraulis australis* (White) is also a small pelagic schooling fish of the family Engraulidae and widely distributed throughout the temperate waters of the Australian and New Zealand coasts (Arnott and McKinnon 1985; Dimmlich and Ward 2006). This species inhabits a depth range between 31 and 70 m (Froese and Pauly 2019). Juvenile *E. australis* are mainly found in inlets, bays, and estuaries, whereas the adults are distributed in open coastal waters (Ward et al. 2003). It feeds primarily on small copepods and other zooplankton. *Engraulis australis* is an important forage fish and is preyed upon by many aquatic predators such as carnivorous fish, squid, dolphins, and seabirds (Froese and Pauly 2019). This fish is economically important because of its extensive use in the fishmeal industry and for human consumption in various parts of the world (Rowling et al. 2010).

*Sardinops sagax* and *E. australis* are generally similar in appearance (Dimmlich et al. 2004). These two species can form dense and broad schools and are targeted by Australian commercial fisheries (Hoedt and Dimmlich 1995; Savage and Hobsbawn 2015).

The eastern school whiting *Sillago flindersi* McKay is a small schooling fish of the family Sillaginidae and found near the seabed, preferring sandy substrates (Froese and Pauly 2019). *Sillago flindersi* is endemic to Australia and is distributed along subtropical and temperate coastal shelves and estuaries (Froese and Pauly 2019; Gray et al. 2014). This species inhabits a water depth less than 100 m, though the species may be found at depth of 180 m along the eastern and southern Australian coasts (Froese and Pauly 2019; Gray et al. 2014). Juvenile *Si. flindersi* usually occur in shallow waters. The species feeds on small invertebrates such as polychaetes, crustaceans (amphipods, decapods, mysids), and ichthyofaunas (Day 2010; Froese and Pauly 2019) within the benthic zone.

In Australia, the above-mentioned three fish species are underrepresented in research on monogenean infection (ABRS 2009 and Table 1). No research has ever been conducted on *S. sagax* in Australia (Table 1) and only a single study conducted by Williams (1988) on *E. australis* from the Swan River Estuary, Perth, Western Australia (Table 1). Williams (1988) identified a novel mazocraeid monogenean species *Pseudanthocotyloides mamaevi* Williams, 1988 from *E. australis*. Extensive studies were undertaken by Dillon et al. (1985a); Dillon et al. (1985b); Hayward (1996a, 1996b); Rohde et al. (1995); Sandars (1945); Williams (1991); Woolcock (1936); Young (1969, 1970) in Australia to identify monogenean infection on multiple sillaginid (whiting) fish species (Table 2) however, *Si. flindersi* received little attention. For example, Hayward (1996b) and Rohde et al. (1995) performed only two studies on *Si. flindersi* from the waters of Coffs Harbour, New South Wales (NSW), and Lakes Entrance, Victoria, Australia, and yielded a single monogenean species, *Polylabris sillaginae* (Woolcock, 1936) Dillon, Hargis, and Harrises, 1983 (Microcotylidae), and an unspecified Microcotylidae species, respectively (Table 1).

**Table 1:** Previous reports of Monogenea identified from the Australian sardine *Sardinops sagax*, Australian anchovy *Engraulis australis*, and eastern school whiting *Sillago flindersi*

Host scienetific name	Host common name (Family)	Monogenea	Family of Monogenea	Geographical localities	Reference
*Sardinops sagax* (Jenyns)	Australian sardine (Clupeidae)	*Mazocraes sardinopsi*	Mazocraeidae	South Africa: Off Port Elizabeth	Reed et al. (2012)
*Engraulis australis* (White)	Australian anchovy (Engraulidae)	*Pseudanthocotyloides mamaevi*	Mazocraeidae	Australia: Swan River Estuary, Perth, Western Australia	Williams (1988)
*Sillago flindersi* McKay	Eastern school whiting (Sillaginidae)	*Polylabris sillaginae*	Microcotylidae	Australia: Coffs Harbour, New South Wales; Lakes Entrance, Victoria	Hayward (1996b)
*Sillago flindersi* McKay	Eastern school whiting (Sillaginidae)	Microcotylidae sp.	Microcotylidae	Coffs Harbour, New South Wales	Rohde et al. (1995)

All monogenean species were identified using morphological method only

**Table 2:** Previous records of monogenean species identified from various whiting fish in Australia. Common name of fish was in accordance with FishBase (Froese and Pauly, 2019)

Host	Host common name	Monogenea	Monogenea family	References
*Sillago analis* Whitley	Golden-lined sillago	*Polylabris queenslandensis*	Microcotylidae	Hayward (1996b)
*Sillago analis*	Golden-lined sillago	*Polylabris williamsi*	Microcotylidae	Hayward (1996b)
*Sillago analis*	Golden-lined sillago	*Monoplectanum youngi*	Diplectanidae	Hayward (1996a)
*Sillago analis*	Golden-lined sillago	*Polylabris australiensis*	Microcotylidae	Hayward (1996b)
*Sillago bassensis* Cuvier	Southern school whiting	*Polylabris sp. 2	Microcotylidae	Williams (1991)
*Sillago burrus* Richardson	Western trumpeter sillago	*Polylabris sillaginae*	Microcotylidae	Hayward (1996b)
*Sillago burrus*	Western trumpeter sillago	*Monoplectanum australe*	Diplectanidae	Hayward (1996a)
*Sillago ciliata* Cuvier	Sand sillago	Gyrodactylidae sp.	Gyrodactylidae	Rohde et al. (1995)
*Sillago ciliata*	Sand sillago	*Microcotylidae sp.	Microcotylidae	Rohde et al. (1995)
*Sillago ciliata*	Sand sillago	*Monoplectanum youngi*	Diplectanidae	Hayward (1996a)
*Sillago ciliata*	Sand sillago	*Bivagina sillaginae	Microcotylidae	Young (1970)
*Sillago ciliata*	Sand sillago	*Monoplectanum australe*	Diplectanidae	Young (1969, 1970)
*Sillago ciliata*	Sand sillago	*Polylabris sillaginae*	Microcotylidae	Hayward (1996b)
*Sillago ingenuua* McKay	Bay sillago	*Polylabris sillaginae*	Microcotylidae	Hayward (1996b)
*Sillago lutea* McKay	Mud sillago	*Monoplectanum youngi*	Diplectanidae	Hayward (1996a)
*Sillago maculata* Quoy and Gaimard	Trumpeter whiting	*Polylabris sandarsae	Microcotylidae	Williams (1991)
*Sillago maculata*	Trumpeter whiting	*Polylabris sillaginae*	Microcotylidae	Hayward (1996b)
*Sillago maculata*	Trumpeter whiting	*Polylabris sillaginae*	Microcotylidae	Hayward (1996b)
*Sillago maculata*	Trumpeter whiting	*Monoplectanum australe*	Diplectanidae	Hayward (1996a)
*Sillaginodes punctatus* (Cuvier)	King George whiting	*Microcotyle* sp.	Microcotylidae	Williams (1991)
*Sillaginodes punctatus*	King George whiting	*Polylabris sp. 1	Microcotylidae	Williams (1991)
*Sillaginodes punctatus*	King George whiting	*Polylabris sillaginae*	Microcotylidae	Dillon et al. (1985a)
*Sillaginodes punctatus*	King George whiting	*Microcotyle sillaginae	Microcotylidae	Woolcock (1936)
*Sillaginodes punctatus*	King George whiting	*Microcotyle parasillaginae	Microcotylidae	Sandars (1945)
*Sillaginodes punctatus*	King George whiting	*Polylabris sillaginae*	Microcotylidae	Hayward (1996b)
*Sillaginodes punctatus*	King George whiting	*Bivagina sillaginae	Microcotylidae	Young (1970)
*Sillaginodes punctatus*	King George whiting	*Microcotyle parasillaginae	Microcotylidae	Young (1970)
*Sillago robusta* Stead	Stout whiting	*Polylabris sillaginae*	Microcotylidae	Hayward (1996b)
*Sillago schomburgkii* Peters	Yellowfin whiting	**Polylabris sillaginae	Microcotylidae	Williams (1991)
*Sillago schomburgkii*	Yellowfin whiting	*Polylabris australiensis*	Microcotylidae	Hayward (1996b)
*Sillago schomburgkii*	Yellowfin whiting	*Polylabris sillaginae*	Microcotylidae	Hayward (1996b)
*Sillago sihama* (Forsskål)	Silver sillago	*Polylabris madagascarensis*	Microcotylidae	Hayward (1996b)
*Sillago sihama*	Silver sillago	*Polylabris sillaginae*	Microcotylidae	Hayward (1996b)
*Sillago sihama*	Silver sillago	*Paradiplectanum sillagonum*	Diplectanidae	Hayward (1996a)
*Sillago sihama*	Silver sillago	*Monoplectanum youngi*	Diplectanidae	Hayward (1996a)
*Haletta semifasciata* (Valenciennes)	Blue weed whiting	*Microcotyle odacis*	Microcotylidae	Dillon et al. (1985b)

*The monogenean species name with asterisks mark (*) have been emended/synonymised with *Polylabris sillaginae* by Hayward (1996b)

^**^Five worms belong to *Polylabris sillaginae* identified by Williams (1991) was later relocated into a new species as *Polylabris australiensis* by Hayward (1996b)

Globally, earlier monogenean researches on the three-fish species have used morphological methods only to identify the worms. Previous morphological species identification has created challenges in the accurate identification of Monogenea from fish in Australian waters Rohde (1989b); Rohde and Watson (1985a, 1985b). As a result, the names of the Monogenea genera and species have been changed, revised, and amended multiple times. Previous researchers, Hayward (1996a, 1996b); Rohde (1989b); Rohde and Watson (1985a, 1985b); Williams (1991) concluded that careful consideration should be given before naming and revising monogenean species within the families Mazocraeidae and Microcotylidae based on low morphological variations. There have been no studies in Australia and elsewhere that have used a combination of morphological and molecular methods to classify and describe monogenean species from *S. sagax*, *E. australis*, and *Si. flindersi*. As a result, specific identification of monogenean species from these hosts using a combined morphological and molecular tool is warranted.

The present study aimed to host record investigation of monogenean species from *S. sagax*, *E. australis*, and *Si. flindersi* and to characterise the species genetically based on partial mitochondrial *cox*1 gene to validate their taxonomic and geographic status.

## Materials and methods

### Fish collection

Three fish species, Australian sardine *S. sagax* (*n* = 55), Australian anchovy *E. australis* (*n* = 70), and eastern school whiting *Si. flindersi* (*n* = 122), were purchased from two retail fish markets in Australia. The fish had been caught from two localities, off the coast of NSW and Victoria, Australia. The details of the fish sampling and examination are provided in Table 3. Fish were transported on ice in an insulated box to the Parasitology Laboratory of Charles Sturt University, Wagga Wagga Campus, Australia. Fish from each batch were examined on the day of arrival at the University. The morphological identification of host fish was confirmed using the keys provided by Gommon et al. (2008).

**Table 3:** Occurrence and abundance of monogenean species infecting three species of Australian fish

Fish species (locality and date)	No. of fish examined	Monogenea parasite	No. of fish infected	Range in infected fish	*P* (%)	Total number found	*MI*	*MA*
Australian sardine *Sardinops sagax*, Off the coast of NSW Date: 29–08–2017	19	*Mazocraes australis*	4	1–1	21	4	1	0.21
		Total	4	1–1	21	4	1	0.21
Australian sardine *S. sagax*, Off the coast of VIC Date: 29–09–2018	36	*Polylabris australiensis*	2	1–2	6	4	2	0.11
		Total	2	1–2	6	4	2	0.11
Australian anchovy *Engraulis australis*, Off the coast of NSW Date: 08–09–2017	70	*Mazocraes australis*	44	1–8	63	116	2.64	1.66
		Total	44	1–8	63	116	2.64	1.66
Eastern school whiting *Sillago flindersi*, Off the coast of NSW Date: 29–08–2017	20	*Polylabris sillaginae*	2	1–4	10	5	2.5	0.25
		*Polylabris australiensis*	1	1–1	5	1	1	0.05
		Total	3	1–4	15	6	2	0.30
Eastern school whiting *Si. flindersi*, Off the coast of NSW Date: 23–07–2018	20	*Polylabris sillaginae*	11	1–3	55	19	1.73	0.95
		Total	11	1–3	55	19	1.73	0.95
Eastern school whiting *Si. flindersi*, Off the coast of NSW Date: 29–08–2018	20	*Polylabris sillaginae*	3	1–4	15	6	2	0.30
		Total	3	1–4	15	6	2	0.30
Eastern school whiting *Si. flindersi*, Off the coast of VIC Date: 29–09–2018	32	*Polylabris sillaginae*	1	1–1	3	1	1	0.03
		Total	1	1–1	3	1	1	0.03
Eastern school whiting *Si. flindersi*, Off the coast of NSW Date: 11–10–2018	30	*Polylabris sillaginae*	15	1–5	50	29	1.93	0.97
		*Polylabris australiensis*	2	1–1	7	2	1	0.07
		Total	16	1–5	53	31	1.94	1.03
Total fish examined (***n*** = 247)		Grand total	84	1–8	34	187	2.23	0.78

*NSW* = New South Wales, *VIC* = Victoria, *P* = Prevalence, *MI* = mean intensity, *MA* = mean abundance

### Parasite collection

Individual fish were examined externally for the presence of monogenean and then dissected to remove the gills. The gills were placed in an individual Petri dish containing saline water (35 g of salt in 1000 ml of water). The surfaces of all gills were thoroughly examined under a stereomicroscope (Leica EZ4, China) for the presence of Monogenea. A total of 187 worms belonging to *Mazocraes australis* Timi, Sardella & Etchegoin, 1999 of family Mazocraeidae and *Polylabris sillaginae* and *Polylabris australiensis* Hayward, 1996 of family Microcotylidae were collected from the examined fish. All parasites were recovered from the gills using fine dissection needles with none observed on the external surface of the fish. Monogenea were washed in saline water, counted, and preserved in 70% ethanol for further morphological and molecular analyses.

The overall prevalence, mean intensity, and mean abundance were 34%, 2.23, and 0.78, respectively. The prevalence, intensity, and abundance of infection of Monogenea were highest in the fish sourced from off the coast of NSW. Among the three Monogenea identified, *M. australis* had the highest overall prevalence and mean intensity at 63% and 2.64, respectively from *E. australis*. Infection with *M. australis* on *S. sagax* was much lower and the prevalence and mean intensity at 21% and 1, respectively. The second most abundant species was *P. sillaginae* infecting *Si. flindersi* from the NSW coast, having the highest overall prevalence and mean intensity, at 55% and 1.93, respectively. Infections of *P. sillaginae* from *Si. flindersi* from the Victorian coast was much lower at prevalence and mean intensity of 3% and 1, respectively. *Polylabris australiensis*, although found on two host species (*S. sagax* and *Si. flindersi*), in NSW and Victoria, was the least prevalent monogenean species found in this study. Table 3 shows the infection data of Monogenea identified from the three species of fish in the present study.

### Morphological examination

Mature Monogenea which were not contracted, broken, folded, or twisted were selected for morphological examination. Handling and processing of specimens were carried out according to Gussev (1973, 1985). Initial morphological analyses were conducted using a compound microscope (Upright Motorized Microscope ECLIPSE Ni-E, Nikon, Japan) fitted with a computer screen. Monogenea were initially grouped based on their key morphological traits such as body shape and size; morphology and morphometry of the sucker, haptor, male copulatory organ, and genital atrium; number and organisation of clamp; shape, size, and the number of hamuli according to Agarwal (1988); Dillon et al. (1985a); Gupta and Krishna (1988); Hayward (1996b); Mamaev (1982); Sailaja et al. (2019); Timi et al. (1999); Williams (1991); Woolcock (1936). The characteristics of systematic importance were measured directly with an eyepiece micrometre (BX‐43 Olympus Microscope, Olympus Corporation, Japan). All measurements are in micrometres and are given as the range, followed by the mean in parentheses. A dash (–) indicates that measurements could not be made. The prevalence, mean intensity, and mean abundance of the monogeneans were determined according to Bush et al. (1997).

### Molecular barcoding of host and parasite

A small piece of the host’s muscle tissue and a small piece from each parasite (the same specimens that were used for morphology as described above) was transferred into separate 1.5 ml autoclaved Eppendorf tubes for molecular study. The remaining anterior and posterior regions of the parasites were processed for microscopy and morphological study. DNA was extracted using DNeasy Blood and Tissue Kits (Qiagen, Hilden, Germany), as per the manufacturer’s instructions, and modified (Shamsi et al. 2017) to be eluted in 40 µl of elution buffer. Polymerase chain reaction (PCR) amplification of the fragment of the mitochondrial *cox*1 gene of both hosts and parasite was carried out using the following primer sets. For fish, FishF1 (forward: 5′-TCA ACC AAC CAC AAA GAC ATT GGC AC-3′) and FishR1 (reverse: 5′-TAG ACT TCT GGG TGG CCA AAG AAT CA -3′) were used and for Monogenea, COI-ASmit1 (forward: 5′-TTT TTT GGG CAT CCT GAG GTT TAT-3′) and COI-ASmit2 (reverse: 5′-TAA AGA AAG AAC ATA ATG AAA ATG-3′) were used (Littlewood et al. 1997; Ward et al. 2005). The cycling conditions to amplify the host’s mitochondrial gene was initial 95 °C for 2 min, followed by 35 cycles of denaturation at 95 °C for 30 s, annealing at 54 °C for 30 s, extension at 72 °C for 1 min, and final extension at 72 °C for 10 min. The mitochondrial gene of Monogenea was amplified according to the protocol described in Hossen et al. (2020). An aliquot (3 µl) of each amplicon was examined on a 1.5% w/v agarose gel, stained with GelRed™, and photographed using a gel documentation system.

Representative samples from hosts and parasites were sent to the Australian Genome Research Facility (AGRF), Queensland, Australia, and were subjected to Sanger sequencing using the same primer sets as for PCR. Sequence data including chromatograms were observed initially through Sequence Scanner Software (Applied Biosystems® Genetic Analysers). The sequences were compared with the GenBank database content with BLAST and deposited in GenBank under accession numbers of hosts and parasites, respectively (Table 4). The evolutionary (pairwise) genetic distance was calculated using MEGA v. 10 (Kumar et al. 2016).

**Table 4:** Details of the Monogenea sequences used in the present study to construct the phylogenetic tree based on *cox*1 data

Monogenea family	Monogenea species	Host family	Host species	Geographical origin	GenBank ID *cox*1	Reference
Mazocraeidae	*Mazocraes australis* Timi, Sardella & Etchegoin, 1999	Engraulidae	*Engraulis australis*	Australia: off the coast of NSW	MZ273894–97	Present study with specimens’ number 60, 61, 67, 127
Microcotylidae	*Polylabris australiensis* Hayward, 1996	Clupeidae and Sillaginidae	*Sardinops sagax* and *Sillago flindersi*	Australia: off the coast of VIC and NSW, respectively	MZ273906–08	Present study with specimens’ number 404, 409, 53
Microcotylidae	*Polylabris sillaginae* (Woolcock, 1936) Dillon, Hargis, and Harrises, 1983	Sillaginidae	*Sillago flindersi*	Australia: off the coast of NSW	MZ273898–MZ273905	Present study with specimens’ number 41, 211, 212, 213, 483, 485, 486, 490
Mazocraeidae	*Neomazocraes dorosomatis* (Yamaguti, 1938) Price, 1943	–	–	–	JQ038229*	Unpublished
Mazocraeidae	*Leptomazocraes orientalis* Mamaev, 1975	–	–	–	KU872044*	Unpublished
Mazocraeidae	*Mazocraeoides gonialosae* Tripathi, 1959	Clupeidae	*Konosirus punctatus*		JF773397	Li et al. (2011)
Microcotylidae	*Microcotyle algeriensis* Ayadi, Gey, Justine & Tazerouti, 2017	Scorpaenidae	*Scorpaena notata*	Off Algeria	KX926443	Ayadi et al. (2017)
Microcotylidae	*Microcotyle caudata* Goto, 1894	Sebastidae	*Sebastes inermis*	–	LC472527*	Unpublished
Microcotylidae	*Microcotyle erythrini* van Beneden and Hesse, 1863	Sparidae	*Pagellus erythrinus*	Off France	AY009159	Jovelin and Justine (2001)
Microcotylidae	*Microcotyle erythrini*	Sparidae	*Pagrus pagrus*	Off Spain	MN816014	Víllora-Montero et al. (2020)
Microcotylidae	*Microcotyle isyebi* Bouguerche, Gey, Justine, and Tazerouti, 2019	Sparidae	*Boops boops*	Off Algeria	MK317922	Bouguerche et al. (2019b)
Microcotylidae	*Microcotyle isyebi*	Sparidae	*Boops boops*	Off Spain	MN816018	Víllora-Montero et al. (2020)
Microcotylidae	*Microcotyle visa* Bouguerche, Gey, Justine & Tazerouti, 2019 c	Sparidae	*Pagrus caeruleostictus*	Off Algeria	MK275652	Bouguerche et al. (2019c)
Microcotylidae	*Microcotyle whittingtoni* Víllora‑Montero, Pérez‑del‑Olmo, Georgieva, Raga & Montero, 2020	Sparidae	*Dentex dentex*	Off Spain	MN816010	Víllora-Montero et al. (2020)
Microcotylidae	*Microcotyle* sp. van Beneden, and Hesse, 1863	Sebastidae	*Helicolenus dactylopterus*	Off Algeria	KX926446	Ayadi et al. (2017)
Microcotylidae	*Paramicrocotyle* sp.^a^ Caballero and Bravo-Hollis, 1972	Pinguipedidae	*Pinguipes chilensis*	Off Chile	KJ794215	Oliva et al. (2014)
Microcotylidae	*Bivagina pagrosomi* (Murray, 1931) Dillon and Hargis, 1965	Sparidae	*Sparus aurata*	Off Australia	Z83003	Littlewood et al. (1997)
Microcotylidae	*Polylabris halichoeres* Wang and Zhang, 1998	Labridae	*Halichoeres nigrescens*	Off China	JF505509	Zhang et al. (2011)
Microcotylidae	*Polylabroides guangdongensis* Zhang and Yang, 2000	Sparidae	*Sparus macrocephalus*	–	JQ038230 *	Unpublished
Mazocraeidae	*Kuhnia scombri* (Kuhn, 1829) Sproston, 1945	Scombridae	*Scomber japonicus*	China: 10 localities along the coast of China	KU380080	Yan et al. (2016)
Mazocraeidae	*Kuhnia scombri*	Scombridae	*Scomber australasicus*	Australia: off the coast of VIC	MZ273889	Hossen et al. (2022)
Mazocraeidae	*Pseudokuhnia minor* (Goto, 1894) Rohde, 1985	Scombridae	*Scomber japonicus*	China	KU379830	Yan et al. (2016)
Mazocraeidae	*Pseudokuhnia minor*	Scombridae	*Scomber australasicus*	Australia: off the coast of VIC	MZ273893	Hossen et al. (2022)
Mazocraeidae	*Kuhnia scombercolias* Nasir and Fuentes Zambrano, 1983	Scombridae	*Scomber australasicus*	Australia: off the coast of VIC	MZ273885	Hossen et al. (2022)
Diclidophoridae	*Choricotyle australiensis* Roubal, Armitage and Rohde, 1983 (Outgroup)	Sparidae	*Chrysophrys auratus*	Australia: New South Wales	MT783686	Hossen et al. (2020)

^*^Sequences published in GenBank only (direct submission)

^a^Genus synonymized with *Microcotyle* (Bouguerche et al. 2019a)

*NSW* = New South Wales, *VIC* = Victoria; all available sequences in GenBank for the species belonging to the families Mazocraeidae and Microcotylidae were included during the construction of the phylogenetic tree

### Phylogenetic analysis

The phylogenetic tree was constructed from the sequences obtained in this study for the mitochondrial *cox*1 region along with available GenBank sequences (Table 4). All available sequences in GenBank for the species belonging to the families Mazocraeidae and Microcotylidae were included during the construction of the phylogenetic tree. All sequences were then aligned with MUSCLE in MEGA v. 10 (Kumar et al. 2016) and manually adjusted. The phylogenetic relationships among the species were inferred using the Bayesian method (MrBayes v 3.2) (Ronquist and Huelsenbeck 2003). The General Time Reversible with Gamma distributed (GTR + G) model was used during Bayesian phylogenetic analysis. This model was proposed by jModelTest v.2.1.4 under Bayesian Information Criterion (BIC) as the best-fit model of nucleotide substitution (Darriba et al. 2012). *Choricotyle australiensis* Roubal, Armitage and Rohde, 1983 (sequence ID: MT783686) identified from Australian waters was used as the outgroup based on close as possible to the species identified (ingroup) in this study (Hossen et al. 2020). The sampling frequency was set at 1000 and the run length continued for 2,000,000 generations until the *p* value reached less than 0.01. After the Markov Chain Monte Carlo (MCMC) run, the first 30% samples were excluded through the ‘burnin’ command. The ‘sumt’ command was executed to summarise the tree. The phylogenetic tree was visualised in FigTree v 1.4.3 (Rambaut 2014).

## Results

### Molecular identification of fish

The preliminary identification of the host species using morphological characteristics was confirmed by the sequencing of mitochondrial cytochrome *c* oxidase subunit I gene (*cox*1). A search in GenBank for the representative sequence generated for *S. sagax* (sequence ID: MZ274053–54) in this study showed 100% similarity with *S. sagax* (sequence ID: DQ107708) identified from the Australian waters (Ward et al. 2005), thus confirming the host’s taxonomic status. The sequences obtained for *Si. flindersi* (sequence ID: MZ274055–56) showed 99% similarity (654/655 with no nucleotide gap) with *Si. flindersi* (sequence ID: EF609468) identified from the Australian waters (Ward and Holmes 2007). In the present study, the exploration of genetic data for *E. australis* was unsuccessful due to the poor quality of the chromatograms.

### Morphological identification of Monogenea

Microscopic examination (which included morphological, morphometric, and meristic data analyses) revealed three species of Monogenea, *Mazocraes australis* of family Mazocraeidae, and *Polylabris sillaginae* and *Polylabris australiensis* of family Microcotylidae.

The morphology and measurements of the monogenean species recovered in this study matched with earlier descriptions given by Dillon et al. (1985a); Hayward (1996b); Timi et al. (1999); Williams (1991); Woolcock (1936) in studies of fish from Australian waters and elsewhere (Tables 5 and 6).

**Table 5:** Comparative measurements of *Mazocraes australis*

Source	Present study	Timi et al. (1999)*
Monogenea	*Mazocraes australis*	*Mazocraes australis*
Host (scientific name)	*Sardinops sagax* and *Engraulis australis*	*Engraulis anchoita*
Host (common name and family)	Australian sardine (Clupeidae) Australian anchovy (Engraulidae)	Argentine anchovy (Engraulidae)
Locality	Australia: Off the coast of New South Wales	Argentina: Coastal area of Buenos Aires Province, Argentine Sea
No. of specimens	Fifteen (*n* = 15)	Ten (*n* = 10)
Total body length (included peduncle and haptor)	1400–2300 (1771)	2,060–2640 (2330)
Maximum body width	150–400 (257)	400–620 (480)
Haptor (opisthaptor) length	190–325 (221)	–
Haptor (opisthaptor) width maximum	150–300 (192)	–
Peduncle length	100–250 (165)	–
Oral sucker length	30–40 (35)	31–40 (36)
Oral sucker width	26–37 (31)	27–36 (32)
Pharynx length	45–60 (54)	55–63 (59)
Pharynx width	35–50 (43)	42–59 (51)
Clamps number	8	8
Largest clamp (anterior pair) length	28–40 (35)	49–55 (51)
Largest clamp (anterior pair) width	38–45 (42)	82–90 (86)
Smallest clamp (posterior pair) length	25–35 (28)	25–29 (28)
Smallest clamp (posterior pair) width	30–38 (31)	29–38 (32)
Genital atrium length	23–35 (26)	25–38 (31)
Genital atrium width	26–38 (30)	27–40 (33)
Number of genital atrial hooks	14–16	16–18
Distance genital atrium–anterior end	150–225 (193)	–
Distance vitellaria–anterior end	225–275 (250)	–
Large hamulus length	36–38 (37)	19–23 (21)
Small hamulus (marginal hook I) length	09–25 (14)	09–15 (13)
Small hamulus (marginal hook II) length	05–08 (06)	06–08 (07)
Egg length (without filament)	200–213 (206)	210–230 (220)
Egg width	50–75 (63)	60–80 (70)

Measurements of *Mazocraes australis* in Timi et al. (1999) have been converted into micrometres

**Table 6:** Comparative measurements of microcotylid Monogenea found in the present study. Measurements are in micrometres and indicated as the range followed by the mean

Source	Present study	Dillon et al. (1985a)	Williams (1991)	Hayward (1996b)	Present study	Hayward (1996b)
Monogenea	*Polylabris sillaginae*	*P. sillaginae*	*P. sillaginae* (syn. *P. sandarsae*)	*P. sillaginae*	*Polylabris australiensis*	*P. australiensis*
Hosts (scientific name)	*Sillago flindersi*	*Sillaginodes punctatus*	*Sillago maculata*	*Sillaginodes punctatus*	*Sardinops sagax* and *Sillago flindersi*	*Sillago schomburgkii*
Hosts (common name and family)	Eastern school whiting (Sillaginidae)	King George whiting (Sillaginidae)	Trumpeter whiting (Sillaginidae)	King George whiting (Sillaginidae)	Australian sardine (Clupeidae) and Eastern school whiting (Sillaginidae)	Yellowfin whiting (Sillaginidae)
Locality	Australia: off the coast of NSW	Australia: off the coast of SA and WA	Australia: off the coast of WA	Australia: off the coast of SA	Australia: off the coast of NSW and VIC	Australia: Shark Bay, WA
No. of specimens	Fifteen (*n* = 15)	Twenty (*n* = 20)	Nine (*n* = 9)	Ten (*n* = 10)	Five (*n* = 5)	Ten (*n* = 10)
Total body length	1325–2725 (2002)	2100–3060 (2410)	992–1568 (1,194)	1350–1,970 (1640)	1300–2650 (1880)	980–2250 (1730)
Maximum body width	250–700 (440)	410–810 (520)	288–352 (320)	430–630 (530)	275–570 (390)	260–540 (420)
Haptor (opisthaptor) length	480–978 (768)	810–1380 (990)	272–544 (398)	1000–1300 (1140)	650–1325 (985)	620–1220 (910)
Oral sucker length	75–88 (82)	52–63 (57)	32–67 (46)	64–78 (71)	63–80 (72)	67–88 (77)
Oral sucker width	45–70 (59)	35–54 (43)	–	–	45–65 (54)	34–52 (43)
Pharynx length	36–50 (44)	36–42 (38)	20–35 (26)	35–39 (37)	40–45 (42)	24–39 (35)
Clamps number (pairs)	27–40 (32)	22–34	21–25 (22)	27–39 (32)	20–35 (29)	19–36 (28)
Clamp length/height (large)	40–55 (50)	40–45 (47)	48–62 (56)	39–49 (44)	42–55 (48)	53–62 (58)
Clamp width (large)	68–80 (75)	59–74 (68)	59–77 (68)	71–80 (76)	65–82 (73)	82–95 (88)
Male copulatory organ length	38–57 (52)	42–54 (50)	45–49 (46)	38–51 (43)	65–70 (68)	64–71 (68)
Male copulatory organ width	20–28 (24)	21–24 (22)	15–21 (19)	20–23 (21)	25–28 (26)	21–27 (24)
Distance vitellaria-anterior end	275–410 (337)	–	–	–	225–325 (267)	–
Egg length (without filament)	–	–	137	163–171	–	192
Egg width	–	–	86	80–89	–	97

*NSW* = New South Wales, *VIC* = Victoria, *SA* = South Australia, *WA* = Western Australia

### Mazocraeid monogenean

#### Mazocraes australis

Based on 15 whole-mount specimens, NSW waters (Table 5). Body elongated, fusiform, or lanceolate. Maximum width near middle and tapering to narrow anterior and posterior region at haptor. Buccal suckers separated. Oesophagus oval. The genital atrium mazocraeid-type and armed with one pair of large lateral hooks and 12–14 small median hooks. Genital hooks organised in two transverse semicircular rows. Follicular vitellaria extending from level of the genital atrium to beginning of peduncle. No vitellaria observed in peduncle and haptor. Haptor well separated from body proper by short peduncle. Haptor heart-shaped and containing eight clamps. Clamps arranged in two opposing rows of four each side. Distance between clamp rows decreased towards posterior region of haptor. Clamps similar in structure. Two anterior pairs larger than remaining posterior pairs in some specimens. Clamp description similar to Timi et al. (1999). Short terminal lappet containing one pair of hamuli and two pairs of marginals. Hamuli strong and stout with hook and moderate ridged shaft. Mature specimens containing spindle-shaped eggs.

### Microcotylid Monogenea

#### Polylabris sillaginae

Based on 15 specimens, NSW waters (Table 6). Body elongated, lanceolate, or fusiform. Two distinct buccal suckers containing sclerotized, tooth-like papillae on rims. Maximum width observed near middle of the body and tapering to narrow posterior region containing clamps. Symmetrical haptor not well separated from body proper. Pharynx and oesophageal diverticula present. Gut bifurcating at or immediately behind the male copulatory organ. Worm bivaginated. Male copulatory organ sclerotised, short, conical, and containing inner tube and outer covering. In relaxed specimens, haptor appears much longer. Clamps organised in two nearly equal ventrolateral rows bearing up to 27–40 pairs in each row. Clamps structurally similar along rows, but differ in size depending on the location, such as anterior, middle, and posterior. Clamp’s description very similar to Dillon et al. (1985a); Woolcock (1936). Vitellaria extends up to intestinal crura.

#### Polylabris australiensis

Based on five specimens, NSW and Victorian waters (Table 6). Body morphology similar to *P. sillaginae*, with an exception in structure of the male copulatory organ. In *P. australiensis*, the copulatory organ comparatively larger and highly sclerotised. Continuous haptor containing 20–35 pairs of clamps observed in elongated and relaxed specimens.

### Molecular characterisation of Monogenea

A total of 16 worms, representing each of the three-monogenean species, were sequenced for *cox*1 gene.

### Mazocraeid Monogenean

Five worms (voucher numbers 38, 60, 61, 67, and 127) belonging to *M. australis*, from NSW *S. sagax* and *E. australis* fish were subjected to sequencing. The genetic sequence for *M. australis* from *S. sagax* (voucher number 38) was failed. The *cox*1 sequences produced for *M. australis* from *E. australis* were 396 bp long and identical. *Mazocraes* species has no *cox*1 sequence data deposited in GenBank that is comparable. As a result, no genetic similarity index was shown for the present specimens. The sequences obtained in this study were deposited in GenBank under the accession numbers (sequence ID: MZ273894–97).

### Microcotylid Monogenea

Eight specimens (voucher numbers 41, 211, 212, 213, 483, 485, 488, and 490) belonging to *P. sillaginae*, recovered from NSW *Si. flindersi* were subjected to sequencing. The length of the *cox*1 sequences generated for the specimens was 396 bp and identical. Three Monogenea (voucher numbers 53, 404, and 409) belonging to *P. australiensis*, obtained from NSW *Si. flindersi* (53) and Victorian *S. sagax* (404 and 409) were subjected to sequencing. The length of the *cox*1 sequences explored for the specimens was 396 bp and identical.

A single *Polylabris* species, *P. halichoeres* Wang and Zhang, 1998 sequence (for the complete mitochondrial gene) is available in GenBank (sequence ID: JF505509) from an unpublished study (Table 4). A nucleotide BLAST search in GenBank for one of the representative sequences obtained from *P. sillaginae* (sequence ID: MZ273898–MZ273905) showed 84% similarity with *P. halichoeres* (sequence ID: JF505509). The sequences obtained for *P. australiensis* in this study did not reveal any similarity with the *P. halichoeres*. The sequences obtained for *P. australiensis* in this study were deposited in GenBank under the sequence ID MZ273906–08.

### Phylogenetic analyses

**Fig. 1:**
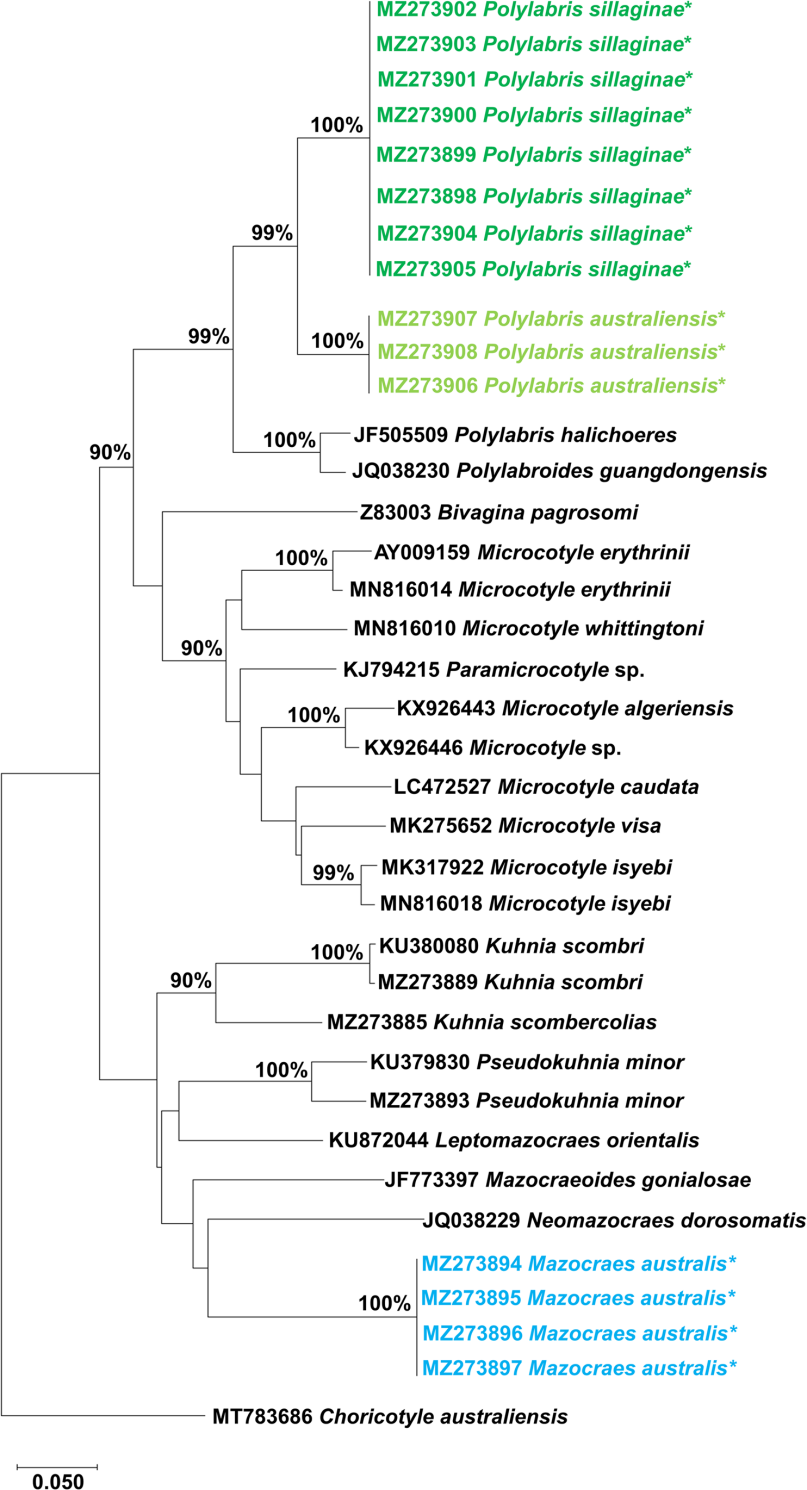
The phylogenetic relationship of Monogenea found in this study and those closely related species available in GenBank (Table 4 for details). The tree has been constructed using Bayesian method. *indicates the *cox*1 sequences generated in the present study. Bayesian posterior probability values more than 90% were shown on the node

Bayesian Phylogenetic tree grouped mazocraeid and microcotylid worms separately. *Mazocraes australis* clustered with closely related mazocraeid species. The sequences obtained in this study for *P. sillaginae* and *P. australiensis* clustered with closely related microcotylid species. The sequences generated in this study for *P. sillaginae* and *P. australiensis* grouped according to their species with a 100% posterior probability value. The phylogenetic relationship of Monogenea found in this study is shown in Fig. 1.

## Discussion

The present study provided for the first-time exploration of *cox*1 sequences of three monogenean species, *M. australis*, *P. sillaginae*, and *P. australiensis*, infecting three Australian commercial fish species. The occurrence of mazocraeid monogenean *M. australis* in two Australian hosts *S. sagax* and *E. australis* is new geographical and host records. The present research also identified two microcotylid Monogenea, *P. sillaginae*, and *P. australiensis*, from two hosts *S. sagax* and *Si. flindersi*; *Polylabris sillaginae* was found in *Si. flindersi*, while *P. australiensis* was found in both host species. In this work, the fish species *S. sagax* was discovered to be a novel host for the monogenean *P. australiensis*. A single earlier study identified *P. sillaginae* from *Si. flindersi* (Hayward 1996b); however, the worm *P. australiensis* was identified for the first-time infecting *Si. flindersi* in this study.

The highest overall prevalence, mean intensity, and mean abundance of monogenean infection were observed in *E. australis* compared to the remaining two hosts *S. sagax* and *Si. flindersi* examined in this study. The monogenean, *M. australis* had the highest overall occurrence and infection (prevalence: 63% and mean intensity: 2.64 (1–8)) and infected two species *E. australis* and *S. sagax* (Table 3). Timi et al. (1999) observed the prevalence and mean intensity of *M. australis* from Argentine anchovy *E. anchoita* Hubbs and Marini at 2.83% and 1.56 (1–9), respectively. The result of the occurrence of *M. australis* in the present study was appreciably higher in Australian waters than observed by Timi et al. (1999) from Argentine waters. There has been no Australian study in which to compare the findings of the present study.

The monogenean, *Polylabris sillaginae* recovered from the *Si. flindersi* was found to be the second most common species in this study, with a prevalence of 55% and a range in infected fish up to 5. Earlier Australian studies observed *P. sillaginae* infection in *Si. flindersi* however, no infection datum was provided (Hayward 1996b). The infection data of *P. sillaginae* found in this study agree with a previous Australian study conducted by Williams (1991) on a sillaginid host, the yellowfin whiting *Si. schomburgkii* Peters. The Williams (1991) study found an infection prevalence of 48% and a range in infected host of 7. The least common monogenean species found in this study was *P. australiensis*. Similar findings were seen for *P. australiensis* in two previous parasitological studies conducted in Australian waters (Hayward 1996b; Williams 1991), though hosts were different (golden-lined sillago *Si. analis* Whitely, and *Si. schomburgkii*).

### Mazocraes species

The genus *Mazocraes* Hermann, 1782 was first proposed by Hermann in 1782 and was later revised by Mamaev (1982). To date, around 14 valid *Mazocraes* species have been identified globally (Table 7). The morphological features, which included morphometric and meristic data of *Mazocraes* monogenean found in the present study, were compared with the valid *Mazocraes* species. Our data showed a complete or partial match with the *M. australis* only, and Table 5 demonstrates the comparative measurements.

**Table 7:** Previous reports of valid *Mazocraes* Hermann, 1782 species identified globally

*Mazocraes* species	Host	Host family	Geographical location	References
*Mazocraes alosae* Hermann, 1782	*Alosa alosa* and *Alosa immaculata*	Clupeidae	Europe (the Black Sea and the Caspian Sea)	Mamaev (1982)
Mazocraes alosae*	*Alosa alosa*	Clupeidae	Western Iberian Peninsula Rivers	Bao et al. (2015)
*M. australis* Timi, Sardella & Etchegoin, 1999	*Engraulis anchoita*	Engraulidae	Argentine Sea	Timi et al. (1999)
M. bengalensis* Sailaja, Shameem & Madhavi, 2019	*Opisthopterus tardoore*	Pristigasteridae	Visakhapatnam coast, Bay of Bengal	Sailaja et al. (2019)
*M. brevoortia* (Hargis, 1955) Mamaev, 1982	*Bravoortia patronus*	Clupeidae	Gulf of Mexico	Mamaev (1982)
*M. chauhani* Kumar and Agarwal, 1981	*Gudusia chapra*	Clupeidae	River Ganges, India	Kumar and Agarwal (1981)
*M. gonialosae* (Tripathi, 1959) Mamaev, 1982	*Gonialosa manmina*	Clupeidae	India	Mamaev (1982)
*M. gussevi* Agrawal and Sharma, 1989	*Hilsa ilisha*	Clupeidae	India	Agrawal and Sharma (1989)
*M. mamaevi* Agrawal, 1988	*Labeo rohita*	Cyprinidae	India	Agarwal (1988)
*M. mehrai* Gupta and Krishna, 1988	*Dussumieria acuta*	Dussumieriidae	Puri, Bay of Bengal	Gupta and Krishna (1988)
*M. multispiralis* Agrawal and Sharma, 1989	*Hilsa ilisha*	Clupeidae	India	Agrawal and Sharma (1989)
*M. sardinopsi* (Lebedev and Parukhin, 1969) Mamaev, 1982	*Sardinops sagax*	Clupeidae	South China Sea	Mamaev (1982)
*M. sprostonai* Gupta and Krishna, 1988	*Tenualosa ilisha*	Clupeidae	Puri, Bay of Bengal	Gupta and Krishna (1988)
*M. stolephorusi* Sailaja, Shameem & Madhavi, 2019	*Stolephorus indicus*	Engraulidae	Visakhapatnam coast, Bay of Bengal	Sailaja et al. (2019)
*M. villelai* (Tandeira and Valdez, 1955) Mamaev, 1982	*Alosa alosa*	Clupeidae	Lisbon	Mamaev (1982)

The *Mazocraes* species name with asterisks mark (*) have the only molecular data available in GenBank

There have been three publications only, which examined *Mazocraes* species genetically and of the 14-valid species globally 12 were identified morphologically. For example, *M. alosae* Hermann, 1782 has seven sequences deposited in GenBank for the nuclear genes only, one sequence for the 18S gene (Bao et al. 2015), and six sequences for the 28S genes (Schade et al. 2016). However, Bao et al. (2015) did not provide any morphological description of *M. alosae*.

A single species *M. bengalensis* Sailaja, Shameem & Madhavi, 2019 has molecular data in GenBank (single sequence deposited for 28S gene) along with a morphological description (Sailaja et al. 2019). However, no *cox*1 data for any *Mazocraes* species is available in GenBank. A nucleotide search in GenBank for one of the four sequences generated in this study showed 79% similarity (306/389; inclusive of 12 nucleotides gap) with another mazocraeid species, *Neomazocraes dorosomatis* (Yamaguti, 1938) Price, 1943 (Sequence ID: JQ038229) from an unpublished study. Therefore, the present study was the first to explore the mitochondrial *cox*1 gene sequences of this monogenean *M. australis*. The phylogenetic tree separated the sequences generated in the present study from the existing mazocraeid species. The strong posterior probability value demonstrated in this study supports the taxonomic status of *M. australis* identification in Australian waters.

The morphological species identification is often difficult, particularly when dealing with small mazocraeid species (Rohde 1989a, b; Rohde and Watson 1985a, b). The following authors concluded that if populations of Monogenea from the same host species or genus occur in different geographical areas, they are likely to be conspecific and should not be classified as different species if they are just slightly different from one another (Rohde 1989a, b; Rohde and Watson 1985a, b). Therefore, seven previously identified *Mazocraes* species are now considered as ‘species inquirendae’ (Sailaja et al. 2019). Further genetic analyses are required to verify the taxonomic status of morphologically identified all *Mazocraes* species.

### Polylabris species

*Polylabris* Euzet and Cauwet, 1967 species are distinguished from other microcotylids by the presence of a single male copulatory organ, which is sclerotised and typically conical (Hayward 1996b). The taxonomic status of species within the genus *Polylabris* is uncertain. Williams (1991) found morphological inter-species variations which had been considered as species novel. As a result, Hayward (1996b) thoroughly revised the genus ‘*with the key to species Polylabris*’ and recognised 17 valid species, as well as three more ‘species inquirendae’ that infected the gills of several Perciformes fish. The morphometric and meristic data of our *Polylabris* worms partially or completely matched with two species, *P. sillaginae* and *P. australiensis* according to the key and diagnostic features provided by Dillon et al. (1985a); Hayward (1996b); Sandars (1945); Williams (1991); Woolcock (1936); Young (1970). The comparative measurements of *Polylabris* specimens are provided in Table 6.

According to Hayward (1996b) and Williams (1991), the taxonomic position of *P. sillaginae* is complicated. *Polylabris sillaginae* was first identified and described from the King George whiting *Sillaginodes punctatus* (Cuvier) sourced from Victorian waters under the name of *Microcotyle sillaginae* Woolcock, 1936. This worm was later recorded from the same host in Western Australian waters and named *Mi. parasillaginae* Sandars, 1945. However, Williams (1991) synonymised the above-mentioned two *Microcotyle* Monogenea as *P. sillaginae* based on their general body forms. In a parasitic study, Dillon et al. (1985a) also identified *P. sillaginae* from the above-mentioned sillaginid host in South Australia and Western Australia. *Polylabris sillaginae* has also been identified from another sillaginid host, *Si. schomburgkii* in Western Australian waters (Williams 1991). However, five of the *P. sillaginae* species identified by Williams (1991) were reidentified as *P. australiensis* (Hayward 1996b). Williams (1991) identified *P. sandarsae* Williams, 1991 from the trumpeter whiting *Si. maculata* Quoy and Gaimard which was described as a novel species based on a few morphological variations (male copulatory organ and testes (shape, size, and number)). Williams (1991) also described *Polylabris* sp. 1 and *Polylabris* sp. 2 from the *Sillaginodes punctatus* and southern school whiting *Si. bassensis* Cuvier, respectively. The names of *P. sandarsae*, as well as *Polylabris* sp. 1 and 2, were later updated and merged into a single species *P. sillaginae* (Hayward 1996b). *Polylabris sillaginae* has previously been found in ten sillaginid hosts in Australia, New Caledonia, and the Gulf of Thailand, with *Sillaginodes punctatus* being the type host (Hayward 1996b). However, in this study, *Si. flindersi* was identified as the host for *P. sillaginae*. For a more comprehensive understanding of *P. sillaginae*, a greater sample size of sillaginid hosts should be examined. Of particular importance is clarification of the specificity of hosts, which according to the findings of the present study requires further investigation.

The general understanding is that monogeneans exhibit high host-specificity and the identification of *Polylabris australiensis* infecting *S. sagax* is an unusual finding of the present study. The name *P. australiensis* was first proposed by Hayward (1996b). So far, this monogenean was identified from two sillaginid fish species, *Si. analis* and *Si. schomburgkii* in Australia (Hayward 1996b). According to Hayward (1996b), earlier morphological characteristic of the species was provided by Williams (1991) under the species name ‘*P. sillaginae*’ identified from the *Si. schomburgkii* in Australia. Williams (1991) observed some morphological differences in some organs such as the size and morphology of male copulatory organs, number of testes, and clamps. Williams (1991) concluded that the morphological discrepancies within the identified *P. sillaginae* ‘*may have been due to inaccuracies of observation and measurement*’ and was ‘*insufficient to separate the worm*’. However, there is no further record of identification of this monogenean species in Australia or elsewhere. The present study confirmed the identification of *P. australiensis* from two new hosts *S. sagax* and *Si. flindersi* in Australian waters. In the present study, the occurrence of *Polylabris* species in *S. sagax* (Clupeiformes: Clupeidae) is uncommon and further research is required to elucidate the reasons for this.

Hayward (1996b) stated that the morphological plasticity of the species belongs to the genus *Polylabris* is high and molecular characterisation would solve the problem of accurate species identification. *Polylabris* species has 11 sequences deposited in GenBank. Nine sequences are available for the nuclear genes and only two sequences for the mitochondrial gene. In particular, *P. halichoeres* has two sequences for the complete mitochondrial gene (Li et al. 2011). *Polylabris sillaginae* has one sequence for the nuclear 28S gene (Catalano et al. 2010) and *P. heterodus* (Lebedev and Parukhin, 1969) Mollaret, Jamieson & Justine, 2000 has one sequence for the 28S (Mollaret et al. 2000). *Polylabris acanthopagri* Mamaev and Parukhin, 1976 has one sequence for 18S, *P. bengalensis* has one sequence for 18S, *P.* cf. *mamaevi* Ogawa and Egusa, 1980 has three sequences for 28S, and an unidentified species *Polylabris* sp. has one 18S and one 28S sequence available in GenBank as a direct submission. However, none of the *Polylabris* species sequences available in GenBank have a morphological description in the publication. A nucleotide search in GenBank for one of the representative *Polylabris* sequences produced in this study revealed 86% similarity with another microcotylid species *Polylabroides guangdongensis* Zhang and Yang, 2000 (Sequence ID: JQ038230) from an unpublished study. Our specimens, however, do not have the similar morphology as *Polylabroides*. The pairwise genetic comparison of the *Polylabris* sequences explored in this study showed 0–10.14% nucleotide variation, which was interpreted as an interspecific variation and confirmed as *P. sillaginae* and *P. australiensis*. The phylogenetic tree divided the *cox*1 sequences generated in this study into two clusters, one for *P. sillaginae* and another for *P. australienis* with a 100% posterior probability value. The tree also separated the *Polylabris* sequences from the existing microcotylid Monogenea sequences with a strong posterior probability value and confirmed the taxonomic status *P. sillaginae* and *P. australienis* (Fig. 1). Further genetic studies are required to confirm the taxonomic status of all *Polylabris* species that have been morphologically identified and described.

## Conclusion

In the present study, three monogenean species *M. australis*, *P. sillaginae*, and *P. australiensis* were identified using morphological and molecular tools. *Mazocraes australis* had the highest overall prevalence, intensity, and abundance among the identified worms. A new host record was established in this study for two monogenean species *M. australis* and *P. australiensis*. The exploration of *cox*1 genetic sequences of these monogenean species are novel in this study. This research has highlighted that populations of Monogenea from the same host genera in different geographic areas are likely to be conspecific and should not be considered as novel species unless both morphological and genetic analyses are performed. To better understand and confirm the taxonomic status as well as geographical distribution of all *Mazocraes* and *Polylabris* species, a broader host examination is required to collect monogenean species and to identify them using combined morphological and molecular analyses.

## Data Availability

The sequences generated for the mitochondrial cytochrome *c* oxidase subunit 1 (*cox*1) gene in this study have been deposited in GenBank database under the accession number MZ273894–97 for *Mazocraes australis*, MZ273898–MZ273905 for *Polylabris sillaginae*, and MZ273906–08 for *Polylabris australiensis*, MZ274053–54 for *Sardinops sagax*, MZ274055–56 for *Sillago flindersi*.
